# The Importance of Chemical Analysis in Cases of Obscure Diagnosis

**Published:** 1879-03

**Authors:** C. A. Doremus

**Affiliations:** Prof. of Chemistry in the Buffalo Medical College


### Medical and Surgical Journal.

Vol. XVIII. MARCH, 1879. No. 8.

### Original Communications.

———:o:———

ART. I.—*The Importance of Chemical Analysis in Cases of Obscure Diagnosis.* Read before the Buffalo Medical Association. By C. A. Doremus, M. D., Ph. D., Prof. of Chemistry in the Buffalo Medical College.

The termination of two of the most exceptional criminal trials ever brought to public notice, and their wide-spread notoriety, has led me to review some of the points especially interesting to the medical profession, and to call attention to the capabilities of medical science in answering certain questions that were presented before the juries. I refer to the trials of the Rev. Geo. B. Vosburgh, for an attempt to poison his wife by administering antimony as tartar emetic, and that of Kate M. Cobb, for the murder of her husband by arsenical poison.

In the early part of 1878, a physician called upon Prof. R. Ogden Doremus, and requested that an analysis of certain fluids be made for the detection of poison. These fluids were tea, medicine and ice-water. The physician stated that he was unable to account for the symptoms in his patient, a lady—he mentioned no name at that time—and he feared an attempt was being made to poison her. Part of these fluids had been partaken of, and vomiting had resulted. No particular poison was suspected.

He was advised to remove his patient at once, and thereby prevent any further dosing—also to save and forward a specimen of her urine, that it might be analyzed. Such a specimen was received the next day, and the physician was telegraphed to reserve more. His request was complied with. Specimen No. 1 was voided Feb. 21, and measured five fluid ounces. Specimen No. 2 was voided Feb. 24, and measured 16 fluid ounces 3 drachms. The analysis of the suspected liquids revealed the presence of antimony in each. In some, tartaric acid and potassa were also detected.

The chemical report showing that poison existed, action was begun by the lady's brother, and an indictment by the Grand Jury brought Vosburgh to trial before the Court of Oyer and Terminer of Hudson Co., New Jersey. He was tried and acquitted. But the verdict of the jury was not the verdict of the people, and deserted by his wife, his parish disaffected, Vosburgh has left his charge and disappeared. It will be impossible to review the whole case, let me therefore cull from the vast amount of medical and chemical testimony the salient points.

The defence admitted that antimony was found in all the suspected liquids.

The defence admitted that antimony existed in the urine.

The defence claimed that the quantities found in the liquids indicated so large an amount of antimony, that had they been partaken of in the proportions stated in the circumstantial evidence, death must have inevitably ensued.

The defence claimed that a specimen of the woman's urine had failed to show, in the hands of their expert, any antimony.

The defence claimed that the woman's brother had dosed all the suspected fluids, and that Vosburgh was innocent of the crime, and his wife an invalid from the effects of disease solely.

In connection with the question of the quantity of poison administered, a discussion arose as to the "tolerance" of poisons. Expert after expert was called to give his opinion. Few had any knowledge of the subject from personal observation of the symptoms produced by the continued action of antimony.

The burden of evidence seemed to lean to the side of "tolerance," and was supported by the authorities quoted.

Reese, in his journal of Toxicology, p.p. 32-37, separates Habit from Tolerance, and defines the latter : "By this is understood the ability of the system to bear very large doses of certain poisonous substances, in consequence of a morbid condition of the economy, and altogether independent of habit. A good illustration is afforded in the case of tartar emetic. In acute pneumonia, and bronchitis, and in acute rheumatism, immense doses of this substance have been given, not only without producing its usual poisonous effects, but with a positive mitigation of the disease. This practice originated with Tomassini, Rasori and Lænnec, and was formerly very much in vogue under the name of the Italian or contra-stimulant system. * * * But the important fact that such quantities are tolerated only in certain morbid states of the system, is studiously kept back." Other authors make similar statements. In this particular case the defence claimed that Mrs. Vosburgh was suffering from a rheumatic affection, and the prosecution not only admitted this, but claimed that the antimony complicated the symptoms. The tartar emetic had been administered repeatedly, and not only "tolerance" may have existed, but also in some degree, "habit."

But whether "tolerance" existed or not, proof was required of the administration of the poison, and the finding of antimony in potions could not furnish such evidence. Antimony must be detected in the vomit, the fæces or the urine.

The woman's brother had obtained each and every one of the liquids, including two specimens of urine, and the defence having disposed of the attending physician, on the score of ignorance, had only the brother to attack, and they charged him with having added the tartar emetic to each separate liquid before sending it for analysis.

Had vomit or fæces been saved, the presence of any poison therein would have been explained away in like manner. For the purpose in view, the urine offers all necessary evidence except that an analysis of fæces or vomit would perhaps furnish more convincing proof of the quantity administered.

Specimen No. 1 of urine, measured 5 fl. oz., was voided Feb. 21, three days after partaking, as was supposed, the last dose of poison.

Specimen No. 2, measured 16 fl. oz. 3 dr., was voided Feb. 24, six days after. Specimen No. 3, measured from 4 to 5 fl. oz., was voided Mar. 2, between 3 and 4 p. m., twelve days after the last dose of poison, and one day after the bowels had been twice evacuated. No evacuation had taken place prior to this for a period of six weeks and two days, and the first dose of poison was supposed to have been given in January, the last on Feb. 18.

Two different methods were used to determine the quantity of antimony in the first specimens. In No. 1 the antimony was extracted, and then weighed as sulphide. In the other, as antimoniate of antimony. No. 1 contained 0.1098 grs. of tartar emetic by calculation from sulphide, or if 40 ounces of urine were voided per diem, 0.8788 grs. No 2 contained 0.2492 grs. of tartar emetic, or for 40 ounces, 0.60875 grs. Thus the actual amount of antimony found in specimen No. 2, exceeded that in No. 1 by $\frac{12}{100}$ of a grain. But if we assume that as much urine was voided on the first day as on the second, then there would be an excess in No. 1 over No. 2. If calculated at 40 ounces per diem, this excess would be $\frac{27}{100}$ of a grain.

No change had taken place during this time in the state of the patient's bowels, nor had there been vomiting.

The attending physician having been discharged, another was called to take his place. He was informed of the accusation, and upon request, obtained a specimen of urine from the woman, and took it to an expert for analysis. It failed to yield any evidence of the poison. How can this failure be accounted for independently of a false charge of attempt to poison?

1st.—A cessation in the elimination of the antimony from the woman's system before the sample was obtained.

2d.—The amount in the urine too infinitesimal to detect.

3d.—An "intermittence" in the elimination.

4th.—An error in the analysis.

ELIMINATION.

Is antimony always eliminated by the urine?

If eliminated, how long a period is required?

What is the rate of elimination?

May the elimination cease, and antimony still remain in the system?

Some of the questions may be answered, not only in regard to antimony, but also in respect to other metallic poisons.

Since the time of Orfilas, experimental researches on the voiding of metallic poisons by the urine, the fact of these bodies being *generally* found in the urine, has been usually acknowledged. When large doses of the metals, especially arsenic and antimony, have been taken, it is the exception not to find them in the urine. Still this exception, though existing, would not apply in this case, as two specimens of urine had been found to contain antimony.

As regards the period of elimination of antimony, it may be generally stated, as commencing shortly after the ingestion of the poison, and though rapidly eliminated, may continue to show itself for two or three weeks. So much depends, though, upon the condition of the bowels, the skin, &c., that no positive rule can be laid down. The rate is a diminishing one usually, at least, many recorded cases show a gradual decrease.

That a poison may exist in the body, may be eliminated by the urine for some days and then cease to appear, some of the poison remaining in the tissues, is an incontrovertible fact. There are numerous cases of this kind cited by Taylor, Tardieu and others, and chemical analysis has proved again and again, the absence of the metal in the urine, the poison having been administered long enough before death for it to appear in that excretion, and though marked reactions were obtained from the tissues.

Mrs. Vosburgh's bowels had been constipated and unmoved up to the day before specimen No. 3 was collected. The skin was dry, and there was no vomiting from Feb. 19th. The only channel of elimination was that of the urine, and if antimony had been administered, it would have been found, as it was found, in that excretion. But the complexion of matters now changed—the patient had had two movements of the bowels, induced by injections, and a new mode of exit was opened, and the excretion by the kidney may have ceased, either from a total elimination or a change in the channel.

As to the second condition, viz. the presence of an infinitesional amount too small for detection.

No doubt a limit exists in the delicacy of chemical reaction, and especially do we find that in the examination of organic mixtures, bodies often escape even the most accurate analysis. The defence claimed, however, that the methods they employed were exceedingly accurate.

They committed, however, a gross error. In this both the physician and the chemical expert were at fault. To be sure, they both regarded this as a case of false accusation, or a case of blackmail, and the symptoms of the invalid being so obscure that doubt existed about it being antimonial poisoning at all, it was deemed essential to test only a small specimen of urine.

This error in judgment on the part of the physician should at least have been corrected by the chemical expert, for the latter should have demanded more than 4 or 5 oz. of urine, and on failing to get evidence, should have called for a second specimen to satisfy not only himself but the cause of justice. If the object were to prove the absence of antimony, the course pursued was the correct one; if the object were to prove either the presence or absence of antimony the method was eminently fallacious.

The search for a metallic poison in still more an organic poison is not like testing for albumen in sugar. Medical men are already aware that chemical analysis fails to reveal the presence of lead in a small quantity of urine, whereas in a larger quantity of urine it is rarely detected by the same process of analysis. No physician should be satisfied with one testing of the urine with a negative result where a graver suspicion existed that the body sought and not found was the cause of the disorder. Taylor says particularly, after citing cases where no poison was found in the urine, though detected in the tissues, "These facts lead to the conclusion that although the urine is an important channel of elimination, we cannot place absolute reliance on a negative result. The detection of it in this secretion shows that the poison has been taken, but the non-detection of it does not necessarily show that there is no arsenic in the body." p. 39, On Poisons.

With pure solution of the metals we are able to arrive at a definite idea of the delicacy of a chemical test, but with compound organic liquids such as urine, where there is reason to suppose, too, that the metal may exist in some peculiar combination with organic matters, we are forced to proceed with other processes for their detection, and the conditions of the tests are as yet not thoroughly established. The chemist should, therefore, be on his guard, lest he draw hasty conclusions, especially when only a small quantity of urine is placed at his disposal.

The next point opens much room for discussion. Are poisons eliminated constantly, though in a decreasing ratio, or is there an ebb and flow, an intermittence in the elimination. Would a specimen of urine be free of a poison at one time, yet if voided a few hours later show its presence? There is great need of research upon this subject. The medical or toxicological works give very meagre or no information. Many claim that in fact there is an intermittence, and argue also by analogy, from the facts recorded in regard to albumen, sugar and other normal as well as abnormal constituents of the urine. No physician would be satisfied from the absence of casts in one specimen of urine of their entire absence. The repeated examination of urine from day to day in cases of albuminuria or diabetes points conclusively to the accepted doctrine. Thudichum in his Pathology on the Urine, on pp. 407-8, points out very clearly this fact in connection with plumbism and mercurialism, and cites a case of lead poisoning, where potassium iodide was administered, yet no lead found in two analyses, yet at a later period it made its appearance. Both physicians and chemists who have had anything to do with examinations for lead can corroborate this observation. My own experience is decidedly this way.

With regard to mercury. Thudichum says, "Not in all cases of mecurialism has it been possible to detect the metal in the urine. In some cases, the metal appeared in the urine at *intervals*, even where the symptoms had undergone no remission. In other cases, the *reappearance* of the metal in the urine was accompanied by a decided increase in the severity of the mercurial symptoms." Much the same applies to copper. It may be argued that arsenic and antimony differ from these metals, in that they are more soluble and seem to be more rapidly voided by the urine. It is claimed that arsenic is voided as an arsenate. Arsenic acid and phosphoric acid produce isomorphous compounds. Is it unreasonable to suppose that these arsenates may not produce analogous effects to that of albumates in the case of lead or mercury, and that an intermittence might occur? Argue as we may, theorize all we please, the facts can only be established by experimental research.

It is not only in cases of poisoning that such information would be of service, but it is a question of importance in therapeutics. We need not only experimental data, but clinical observation, that the aberration from the normal functioning of the body shall be clearly understood.

But admitting that all the causes so far enumerated to account for the failure to detect antimony do not apply in this instance, there remains still another which would account for the negative result offered by the defence. This lays in an error in the process of analysis.

The urine at this voiding, viz., 12 days after the last dose of poison, could not contain at most much antimony, nor would it be reasonable to expect a decrease by an arithmetical ratio, as the defence absurdly set up. The expert could only hope to find a trace at least. To render the detection *sure*, however, he divided the sample, 4-5 oz., into three unequal portions, the largest being about half, and then applied different tests to each part. Wherein was such a course warranted? Who could expect to get proper evidence using only an ounce or two of urine? In the annalysis of the body of Mr. Cobb it was deemed advisable by the State Attorney that I should have an associate, and it was suggested that the viscera should be divided and each one of us should make an analysis. I protested against such a procedure, and the result of the analysis has abundantly corroborated my judgment. Had certain parts been divided and separately analyzed no poison would have been found, whereas from the larger quantity, clear and unmistakable proof was readily obtained. Woodman and Tidy in their Forensic Medicine and Toxicology, p. 77, in speaking of dividing the parts sent for analysis and searching for various poisons by special tests, conclude by saying, "In the second case, as we need hardly say, the operator would be adopting the best possible means of securing a failure; for if the poison be present in very small quantity, the division of it into many parts would be a certain way of baffling the investigator." But besides dividing the specimen the method of analysis employed upon each part was open to serious objection; so, that taken altogether, the door is left wide open for doubts upon the question of absence of poison. The chemist, on cross-examination, admitted that he was unaccustomed to the analysis of urine or of fæces; did not know the constituents of normal urine, and was unfamiliar with some of its normal properties. He, notwithstanding; descended to a personal attack upon the expert for the prosecution, and belittled himself and science in decrying some of the most refined methods of chemical research. Under all the circumstances then, the prosecution had reason to believe that the presence of antimony in Mrs. Vosburgh's urine was demonstated. The defence in addition had failed to save the fæces and have them analyzed, and had made no report of an analysis of a pocket lining said to contain antimony. This antimony was spilled from a vial in Sickles' pocket, and the truthfulness of important evidence hung upon the presence of tartar emetic in the lining.

The medical evidence on the symptomatology of the case was conflicting in the highest degree. The physicians called by the defence were inclined to judge the case from a medical standpoint only, and to ignore the value of the urinary examination. Those for the prosecution in addition to testifying that the symptoms might be accounted for by antimonial poisoning, as well as "rheumatoid gout of the stomach," as the defence set up, considered the chemical analysis as the deciding point—the pivot upon which the whole case turned.

They scouted the idea that a country farmer could have dosed the specimens of urine so that they should contain only that trace of poison found, and do it so scientifically as to show the relation that existed in regard to decrease in elimination. They saw abundant reasons, however, from many standpoints to account for the failure to detect antimony in the specimen of urine sent to the expert for the defence. The judge charged the jury that antimony was found. It remained for them to decide whether Vosburgh had administered it or not. Their verdict was as I stated, an acquittal, but the villainous character of part of the circumstantial evidence and the subsequent discovery by a druggist, at whose store Vosburgh was a frequent visitor, that about an ounce of tartar emetic had been stolen from his shelf, coupled with the druggist's recollection of conversations with Vosburgh on the subject of poisons, has turned the verdict in the minds of the community, and Vosburgh has been obliged to leave the city and his pastoral charge.

Throughout the trial the necessity of chemical analysis, as an adjunct to diagnosis, is especially made evident. The necessity, too, of searching scientific investigation upon the absorption and elimination of poisons was clearly demonstrated.

The next case I propose calling attention to, is that of the demise of Chas. H. Cobb, Jr., of Norwich, Conn., under suspicious circumstances. Enjoying general good health, in the early part of February, 1878, Mr. Cobb was seized with an unaccountable attack of sickness, accompanied by strange symptoms. His medical adviser gave him some relief, but subsequently Mr. Cobb was again violently attacked, and presented, among others, symptoms of partial paralysis. This was attributed to lead poisoning, and he was treated accordingly. Nevertheless the case was obscure and the diagnosis very unsatisfactory. Though prescribed for, his general health became worse, and on June 5th, soon after a meal, he was seized with convulsions and died suddenly, with opisthotonos that was ascribed to strychina.

The viscera were sent to me for chemical analysis, and the search for poisons revealed arsenic and copper. Testimony to this effect in connection with circumstantial evidence, induced the Coroner's jury to order the examination of the body of Mrs. Hattie E. Bishop, also of Norwich, and at my request the body of Chas. H. Cobb, Jr., was also disinterred.

Mr. Bishop and Mrs. Cobb were confined in jail to await further developments.

Mrs. Bishop's body also contained arsenic and copper.

I had obtained, on the exhumation of Cobb's body, the greater part of the brain, a portion of thigh muscle, and about four or five ounces of urine that remained in the bladder. I am confident from the condition in which I found the brain and urine, that there was no post-mortem imbibition. None of the viscera were present, and there was only a little exudation of liquid in the abdominal cavity. The brain placed in the abdomen after the first post-mortem did not touch this fluid. The urine was bloody in color, and already partly decomposed.

On chemical analysis, the urine gave evidence of arsenic, and about 0.06 of a grain of arsenious sulphide, equal to 0.05 of a grain of arsenious oxide was extracted.

I was impressed by the approximation in the amount of arsenic in this case, to the amount of antimony in the other. In the manipulation of the two about equal amounts of urine were examined, and the precipitates of antimony and arsenic appeared about corresponding in bulk. The amounts of antimony and arsenic partaken in these cases respectively, and the period of time over which they were being absorbed, I should judge, from the circumstantial evidence, to be nearly equal. Arsenic was by this analysis of the urine, then, proved to have been administered some time previous to death. Only a little was found in the contents of the stomach and intestines and brain, while larger amounts were extracted from the urine, spleen and kidneys. Arsenic was thus demonstrated to have been absorbed.

In this trial the defence claimed that Cobb was an arsenic eater, and that "Habit" or "Tolerance" *could* be established. It would seem, then, comparing these two cases of poisoning, as if "salvation to the one was death to the other." Mrs. Vosburgh could not have "tolerated" the amount of poison administered and live. Mr. Cobb died because he stopped taking the poison. The science of Toxicology would be grateful if the medical profession could decide speedily upon the question of "tolerance" and "habit." The *facts* adduced by the chemist after months of laborious search, are too frequently explained away by some theorist in medicine.

You are all familiar with the termination of this trial. Mrs. Cobb has just been convicted of murder in the second degree, and sentenced to a life imprisonment. Mr. Bishop has confessed to the double crime and will be tried shortly.

But this case of poisoning seems without parallel. According to Bishop's confession, his own wife was murdered probably by arsenic given by Mrs. Cobb, and morphia administered by himself. Arsenic I have already found, but have not, as yet, concluded the search for morphia.

Mr. Cobb was dosed, first with aconite, secondly by morphia, then arsenic and finally strychnia. Neither aconite nor morphia could be found—they were administered too long before death to be detected. But the analysis did demonstrate arsenic and strychnia, and besides these, copper and bismuth. The copper came probably from many sources, and was proved to be unconnected with the arsenic. Bismuth had been prescribed as a medicine.

Mr. Cobb had partaken of strychnia in medicine for a couple of days before his death, to the amount of one-half a grain in all—but this would scarcely account for its detection in the contents of the stomach, intestines and in the liver.

During this trial the question of absorption and elimination also came up, in addition to that of "tolerance," and the same dearth of facts was as painfully apparent as in the Vosburgh trial.

Having before us the main features of these two cases so diametrically opposed when viewed from one stand-point, so wonderfully corroborative when viewed from others, let us draw a few conclusions.

1st. Authorities seem to agree in regard to the habituation of the human system to organic poisons, but claim that an *inorganic* poison cannot be administered in much more than a medicinal dose without danger of its toxic effects. A "tolerance" may exist in some diseased states of the system, but only for special poisons—as with antimony in the treatment of pulmonary affections. Arsenic, most claim, cannot be thus administered.

2d. That both arsenic and antimony are rapidly absorbed, but that there is much need of extended clinical observation in regard to the rapidity of such absorption. The literature is quite comprehensive on this point in regard to arsenic, but not so with antimony.

3d. As regards elimination, few facts are known, and there is great need of thorough systematic research. Under this head the following questions deserve attention:

(a) Is the poison always eliminated? From what is already known we can answer *generally* yes. Sometimes, however, the poison fails to show itself.

(b) Does the elimination continue, having once commenced, till all poison is removed from the system? If by all the channels, undoubtedly yes; but if only by the urine, the record of cases says decidedly no.

(c) Is the rate of elimination continuously decreasing, or does it fluctuate?

(d) Does the fluctuation ever amount to a disappearance with subsequent reappearance?

If the chemist were called oftener to aid the physician in the treatment of cases of accidental or suicidal poisoning, an answer to these two last propositions would soon result.

However problematical the search for poisons may *seem*, these two cases furnish actual proof of the benefit that accrues from a careful chemical analysis. Instruction is now given in the medical schools, with regard to the analysis of the urine, and physicians are familiar with the tests for abnormal constituents, such as sugar and albumen. The search for poisons is, however, far more intricate, and demands special facilities that the ordinary practitioner does not possess, and requires more time than he has to spare. He must call, therefore, upon an *expert* chemist. I say advisedly expert, for the processes of ordinary analytical chemistry frequently do not yield results when applied to the examination of organic mixtures. Analyses of this kind become almost a specialty, and demand an acquaintance with medicine which is wanting, as a rule, in chemists.

When a thorough analysis is made, however, the poison, if in the system, will *generally* be found, for it is *generally* eliminated. Its detection is *sure* proof of its administration, and a positive indication of the cause of the disorder. Only after a negative result with two or more samples of urine are we justified, however, in claiming an *absence* of poison, and even this needs qualification in special cases.

The analysis of the suspected liquids and of the urine, was with Mrs. Vosburgh the means of recovery. A timely stoppage of the dosing, a prompt administration of suitable and nutritious food raised her from the sick bed. A thorough analysis of Cobb's urine would have undoubtedly revealed the presence of arsenic, and been the means of saving his life. It is not among the low-bred and the vulgar that we meet with these dastardly attempts against human life, the record of cases, especially with antimony, shows that the perpetrators of such crimes live in a higher sphere of society. It is, therefore, doubly necessary for the physician to be watchful, and when he mistrusts, or when the disease baffles his powers of diagnosis, he may, I claim, find ready help at the hands of the chemist.

NOTE.—While the above was in press my attention was called to a note published by the expert for the defence in the Vosburgh case in the Proceedings of the American Chemical Society. Vol. II, No. 4, p. 144. After giving the results of several analyses he remarks. "The above observations and results would indicate that antimony, when swallowed even in very small doses, is very promptly eliminated, in part by the kidneys, and continues to be so eliminated constantly. With such small doses as were here employed, (one grain and a half in three days), it is not likely that the elimination could have been traced much further than it was in these experiments, namely, for five days after the last dose." It is beyond the bounds of the purpose of my present article to criticise the chemistry of the method of analysis. That I reserve as a fruitful field of future work.

For the present I will only call attention to the fact, that in the analyses of urine referred to in the above extract never less than 7¼ fl. oz., and sometimes as much as 30 fl. ounces were taken for analysis. Yet this same expert was willing to stake his scientific reputation on an analysis of 2½ fl. oz. of urine *twelve* days after the administration of the last dose of tartar emetic.

